# Spectrum of Perceptions Regarding Professional Climate: A National Survey of U.S. Cardiologists and Trainees

**DOI:** 10.1016/j.jacadv.2025.102340

**Published:** 2025-11-15

**Authors:** Kevin L. Thomas, Laxmi S. Mehta, Anne Rzeszut, Sharonne Hayes, Jennifer H. Mieres, Melvin Echols, Andrew P. Miller, Michelle N. Johnson, Garima Sharma, Pamela S. Douglas

**Affiliations:** a Duke Clinical Research Institute and Division of Cardiology, Duke University School of Medicine, Durham, North Carolina, USA; b Division of Cardiology, The Ohio State University Wexner Medical Center, Columbus, Ohio, USA; c American College of Cardiology; d Department of Cardiovascular Medicine, Mayo Clinic, Rochester, Minnesota, USA; e Department of Cardiology at Northwell, Zucker School of Medicine, Lake Success, New York, USA; f Division of Cardiovascular Disease, University of Alabama at Birmingham, Birmingham, Alabama, USA; g Division of Cardiology, Memorial Sloan-Kettering Cancer Center, Association of Black Cardiologists’ Representative, New York, New York, USA; h Division of Cardiology, Inova Schar Heart and Vascular, Falls Church, Virginia, USA

**Keywords:** culture, organizational effectiveness, respect, workplace climate

## Abstract

**Background:**

Characterizations of cardiology’s professional climate often focus on negative experiences.

**Objectives:**

The study’s primary objective was to broadly characterize cardiology climate.

**Methods:**

The American College of Cardiology surveyed 1805 cardiologists online in 2022. Agreement with cardiology 7 workforce statements could be categorized into 4 attitudinal profiles (A-D) in 1513 (84%), using a 2-step cluster analysis maximizing log-likelihood measures of agreement. Multivariable modeling described respondents’ alignment with profiles. ORs compared perceptions across profiles.

**Results:**

Most respondents were men (n = 1,095, 72%), heterosexual/cisgender (n = 1,380, >90%), and identified as White (n = 800, 53%). Profile A (441, 29%) perceived cardiology’s climate as empowering/inclusive/no change needed. Profile D (n = 278; 18%) felt climate was stifling/exclusionary/change needed. Intermediate were B: inclusive/change needed (n = 501; 33%) and C: stifling/uncertain about change (293; 19%). Profile A was independently associated with male sex, White race, late career, and no mistreatment, C-statistic = 0.73 (95% CI: 0.70-0.75). Profile D was associated with female sex, Black, Asian, Hispanic or other race individuals, early/mid-career, and experience of mistreatment, C-statistic = 0.80 (95% CI: 0.77-0.83). Men were respected by most (93%; range across profiles 90%-96%) with less agreement about people identifying as Black, Asian, Hispanic, or other (68%; 27%-90%); women (68%; 25%-95%); people with a disability (54%; 22%-77%); and nonheterosexual (48%; 19%-69%) or transgender/sex nonconforming people (23%; 5%-36%). Primary workplace culture was perceived more positively, while organizations were seen as ineffective in improving climate.

**Conclusions:**

Cardiologists’ perceptions of professional climate differ widely with 37% viewing it as stifling/exclusionary and 51% desiring change. These findings, plus perceived organizational ineffectiveness, support efforts to improve the cardiology workplace climate.

Like most professions, cardiology aspires to have a positive culture. However, the climate, or “shared perceptions of organizational policies, practices, and procedures” may not align well with these high aspirations despite concerns about ensuring clinician well-being.^1^^,^^2^ Instead, discrimination and mistreatment are common in cardiology globally and across the workforce,^3^, ^4^, ^5^ beginning with residents who cite a negative culture as a primary reason not to specialize in cardiology.^6^^,^^7^ While negative incidents are relevant and reflect culture, direct assessment of both positive and negative views provides a more holistic perspective. Fully understanding the lived experiences of cardiologists and trainees is essential to ensure workforce well-being and optimize patient care and outcomes.^2^^,^^8^^,^^9^

Unfortunately, there are few studies of the broader cardiology climate. Studies in other fields consistently find that experiences of culture, or climate, vary by demographic and other personal characteristics. For example, a multispecialty survey of prior National Institutes of Health K grant awardees noted significantly less positive views of general climate overall by women as compared to men, with no by race and ethnicity or LGBQT + status.^10^ Similarly, an analysis of residents found substantial variation in vitality by specialty, with high vitality ranging from 15% to 70% with up to 30% being dispirited.^11^ Dissatisfaction with professional climate is not unique to medicine. Other professions have performed surveys, including the American Economic Association, whose 2019 survey noted that only 34% of respondents were “satisfied with the overall climate within the field of economics” with responses varying by age, sex, disability, religion, and sexual orientation.^12^

Thus, the professional climate and lived experiences of U.S. cardiologists and trainees cannot be imputed from existing data; prospective study over a full range of demographic groups is required. To this end, we deployed a national survey asking cardiologists and cardiology trainees about perceptions of the cardiovascular professional climate. In contrast to a more traditional variable-centered approach relating specific characteristics to particular experiences, we used a person-centered approach to identify attitudinal profiles based on multiple indicators and then examined the characteristics of those aligned with each.^13^

## Methods

The American College of Cardiology (ACC) conducted a 31-item online survey of U.S. cardiologists and fellows-in-training (FITs) (“cardiologists”) in 2022. Survey questions were adapted from prior ACC surveys,^3^^,^^4^^,^^14^ Workplace Incivility Scale,^15^ National Institutes of Health Culture Climate Survey,^16^ and American Economy Association’s Professional Climate Survey.^12^ A single-item measurement question from the mini-Z survey assessed burnout.^17^ Patient Health Questionnaire-2 and Generalized Anxiety Disorder 2-item screening tests assessed depression and anxiety.^18^^,^^19^ The full survey is available at https://doi.org/10.1016/j.jacadv.2025.101666 and the results for workplace incivility have been reported.^5^ This study follows Strengthening the Reporting of Observational Studies in Epidemiology (STROBE) reporting guidelines.

Demographic information was self-reported and included age, sex identity, sexual orientation, race and ethnicity, career stage, immigration, burnout, and experiences of mistreatment with related career impact. Questions focused on agreement with statements about professional climate in 4 areas: cardiology workforce related to diversity (as defined by the respondent), respect, primary workplace climate and workplace effectiveness, and included free-text comments on diversity, inclusion, and discrimination. Within each area, statements were presented in random order to avoid influencing subsequent answers. Advarra Institutional Review Board Services determined the research project exempt from oversight per DHHS 45 CFR 46.104(d) (2).

### Survey deployment

The survey link was emailed to 18,234 U.S. cardiologists and FITs from the ACC database of 29,277 U.S. members, utilizing a random sampling stratified by race and ethnicity and was open from March 4, to May 16, 2022. A deidentified random drawing for 4 $250 Apple gift cards incentivized participation.

### Profile creation

1805 respondents completed the anonymous survey (10% response rate; similar to other recent ACC surveys).^3^, ^4^, ^5^ Demographics of included respondents and nonrespondents were similar (data not shown). 1513 cardiologists (84%) answered all statements required for classification into profiles by cluster analysis and formed the study population. Profiles were determined by analyzing patterns of agreement with 7 workforce statements (Question 1 in survey) in a two-step, cluster analysis approach to identify homogeneous segments within the data set (Supplemental Figure 1). This method was chosen for its ability to handle categorical and continuous variables and efficiency with large sample sizes. Multiple approaches to developing the clusters were explored; based on preliminary analysis, inclusion of 4 profiles yielded the best fitting model with the greatest discrimination between clusters. We opted to select this approach rather than allowing the algorithm to select the number of clusters. Hierarchical log-likelihood measures were used to identify profile inclusion for each individual that resulted in a silhouette coefficient of 0.4 indicating fair cohesion and separation (Supplemental Figure 2). Cluster analysis is particularly suited to uncovering complex relationships, modeling differences, and understanding individual heterogeneity.

### Statistical analysis

Comparisons between groups were determined using chi-square tests. Univariate logistic regression determined the associations of profile B, C, and D responses with perceptions of profile A. Multivariable logistic regression models identified independent predictors of assignment to profiles. Thresholds for variable model entry and for staying in the model were *P* < 0.01 to account for multiple comparisons. Entered variables included sex, sexual orientation, career stage, living full time in the United States, disability, race, experiencing workplace mistreatment, anxiety, depression, and burnout. Free-text entries were categorized by using QDA Miner software, Provalis Research. Profile creation and analyses of all response data were performed using IBM SPSS Statistics for Windows Version 25.0.

## Results

Of the 1513 respondents included in the profile analysis, 72% identified as men, 25% as women, 1 individual identified as nonbinary, and 3% did not wish to disclose their sex identity (Table 1). Over 90% reported their sexual orientation as cis/straight. Most identified as White race, 53% or Asian race, 23% (including 16% South Asian and 7% East Asian), while 6% identified as Black race, 4% Middle Eastern heritage, and 8% Hispanic ethnicity. Only 2% reported a mental or physical disability. Most participants (64%) were born in the United States and an additional 20% arrived in the United States before or during training. Respondents represented all career stages: 41% had been practicing ≥22 years; 15%, 1 to 7 years; 12%, 8 to 14 years; 11%, 15 to 21 years; and 13% were FITs. Most reported experiencing incivility, harassment or discrimination (77%), and 29% reported feeling burned out.

**Table 1:** Respondent Demographics and Personal Characteristics Overall and by Profile

	All Respondents	Profile A	Profile B	Profile C	Profile D	*P* Value for Intergroup Differences
		Empowering, Inclusive Culture/No Change Needed	Empowering, Inclusive Culture/Change Needed	Stifling, Exclusionary Culture/Change Uncertain	Stifling, Exclusionary Culture/Change Needed	
	(N = 1,513, 100%)	(n = 441, 29%)	(n = 501, 33%)	(n = 293, 19%)	(n = 278, 18%)	
Sex						<0.0.001
Men	1,095, 72%	35%	35%	17%	13%	
Women	378, 25%	8%	30%	28%	35%	
Nonbinary/Agender/Something else	1, 0%	0%	0%	0%	0%	
Do not wish to disclose	39, 3%	64%	15%	15%	5%	
Sexual orientation						<0.0.001
Heterosexual/Straight	1,414, 93%	29%	34%	19%	19%	
Gay/Lesbian	25, 2%	12%	24%	28%	36%	
Bisexual	9, 1%	11%	56%	11%	22%	
Do not wish to disclose	65, 4%	55%	21%	18%	5%	
Race						<0.0.001
White	800, 53%	35%	35%	18%	12%	
South Asian	250, 16%	19%	35%	23%	23%	
East Asian	100, 7%	20%	34%	31%	15%	
Black	96, 6%	4%	22%	13%	62%	
Hispanic	119, 8%	26%	32%	20%	22%	
Middle Eastern	66, 4%	29%	42%	12%	17%	
Declined to provide	82, 5%	45%	16%	23%	16%	
Career stage						<0.0.001
In training	196, 13%	16%	44%	16%	23%	
1-7 y	223, 15%	20%	34%	18%	28%	
8-14 y	187, 12%	25%	32%	17%	27%	
15-21 y	172, 11%	32%	33%	20%	16%	
22+ y	614, 41%	39%	31%	19%	11%	
No training data	121, 7%	31%	35%	19%	15%	
Live full-time in the U.S.						<0.0.001
At birth: born and raised in the U.S.	965, 64%	33%	34%	17%	16%	
Before or during college	141, 9%	26%	28%	21%	26%	
Before or during medical school	13, 1%	8%	31%	54%	8%	
Before or during residency/fellowship	304, 20%	21%	36%	23%	20%	
After completing training	53, 3%	21%	25%	28%	26%	
Other, please specify:	20, 1%	15%	25%	15%	45%	
Do not wish to disclose	17, 1%	50%	17%	33%		
Disability						0.059
Yes	36, 2%	33%	25%	22%	19%	
No	1,438, 95%	28%	34%	20%	18%	
Do not wish to disclose	39, 2%	54%	19%	11%	16%	
Experience discrimination						<0.0.001
No mistreatment	346, 23%	25%	32%	21%	23%	
Yes (any mistreatment)	1,167, 77%	45%	37%	14%	4%	
Workplace incivility	539, 36%	18%	27%	24%	31%	
Discrimination/unfair treatment	975, 64%	23%	30%	23%	25%	
Emotional/physical harassment	500, 33%	19%	27%	24%	29%	
Sexual harassment	211, 14%	10%	22%	24%	45%	
Burnout and psychologic distress						
Burned out^a^	444, 29%	24%	28%	21%	27%	<0.0.001
Anxiety disorder (score = 3-6)^b^	184, 12%	22%	31%	23%	25%	0.016
Depressive disorder^c^ (score = 3-6)	130, 8%	25%	25%	25%	25%	0.021

Values are n, %.

a Data obtained using the mini Z survey.^17^ Column percents are 25%, 25%, 32%, 43%, profiles A through D, respectively.

b Data obtained using the Generalized Anxiety Disorder 2-item (GAD-2) survey.^19^ Column percents are 9%, 11%, 15%, 16%, profiles A through D, respectively.

c Data obtained using the Patient Health Questionnaire-2 (PHQ-2).^18^ Column percents are 7%, 7%, 12%, 12%, profiles A through D, respectively.

### Profile alignment

The 4 profiles derived from Question 1 responses were characterized according to 2 scales, perception of the climate (workforce and respect) and desire for change (Central Illustration). The perceptions of those in profile A (n = 441: 29%) were best described as cardiology having a thriving and inclusive climate with no improvement needed. Profile B (n = 501; 33%) had perceptions of a thriving and inclusive climate with change needed. Profile C (n = 293; 19%) perceived a stifling and exclusionary climate and was uncertain about change. Profile D (n = 278; 18%) felt that cardiology had a stifling and exclusionary climate, and that change was needed. Thus 571 (C + D: 37%) felt the climate was stifling and exclusionary and 778 (B + D: 51%) felt change was needed. Only 29% felt that the cardiology climate should not be improved.

**Central Illustration:**
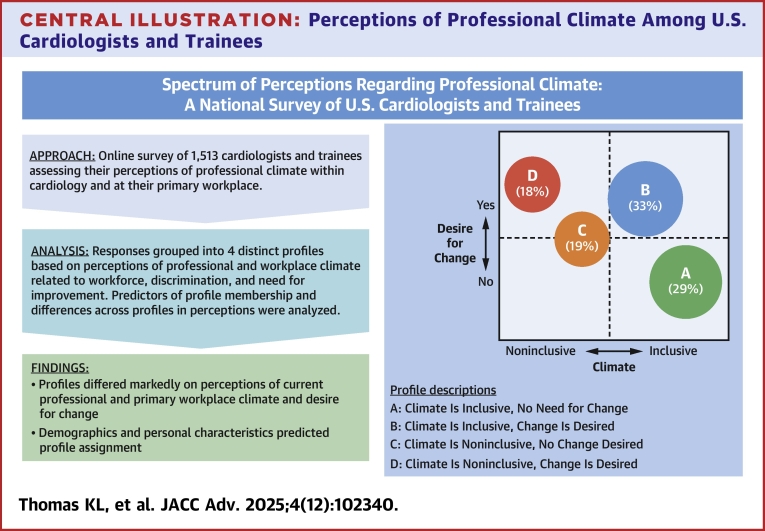
**Perceptions of Professional Climate Among U.S. Cardiologists and Trainees** A survey assessing perceptions of workplace climate among cardiologists demonstrated a spectrum of responses. Two of these are plotted: inclusiveness of climate (culture as experienced) and desire for change.

### Profile characterization

**Figure 1:**
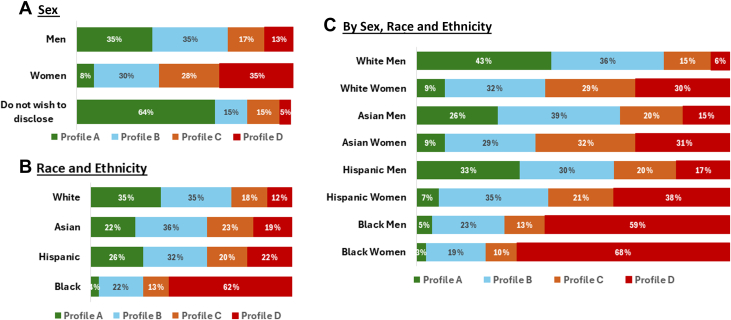
**Profile Demographics by Sex and Race and Ethnicity** Bar graphs show variations in profile membership by sex (A), race and ethnicity (B), and their intersection (C).

Virtually all demographic and identity characteristics varied significantly across profiles (Table 1, Figure 1). The perceptions of most men (70%) were aligned with profiles A or B while most women (63%) aligned with C or D (Figure 1A). Similarly, most White, Asian and Hispanic individuals were aligned with A or B (70%, 58% and 58%, respectively); most Black respondents aligned with D (62%) (Figure 1B). Profile demographics analyzed by both race and ethnicity and sex showed significant intersectionality (Figure 1C) with marked differences in perceptions between men and women of White, Hispanic, and Asian race/ethnicity, but not between Black women and men. Within sex, women of White, Hispanic, and Asian race/ethnicity had somewhat similar perceptions, as did men from these groups, while Black women and Black men each differed substantially from others of the same sex.

Differences between profiles were notable for other characteristics. Higher proportions of older cardiologists and those born in the United States or immigrating before training were in profiles A and B. Personal disability was rare and not associated with profile alignment.

While mistreatment (incivility, discrimination, and/or harassment) was common (77% of respondents), the prevalence varied by profile, with 65% of A respondents reporting mistreatment, and B: 75%, C: 83%, and D: 95%. Similarly, anxiety, depression, and burnout varied by profile with 25% of A respondents feeling burned out, and B: 25%, C: 32%, and D: 43% (Table 1). Specific types of mistreatment were reported 2 to 3 times more frequently by D as compared to A including, among those doing clinical work, delayed professional advancement (55% vs 18%), differing clinical work expectations (43% vs 16%), hiring practices (36% vs 11%), and compensation (40% vs 12%). Among those doing academic work, types of mistreatments more common in D included delayed professional advancement (34% vs 14%), compensation (32% vs 20%), access to research opportunities, and publishing roles (35% and 29% vs 11% and 12%) (Supplemental Table 2). Negative consequences of mistreatment were more common in D, including taking action to avoid harassment or discrimination, feeling silenced and social avoidance (Supplemental Table 3). However, among those experiencing harassment or discrimination, the outcomes were similar.

### Predicting profile alignment

Demographic and identity characteristics predicting inclusion in profile A on multivariable analysis included self-identified male sex, White race, late career stage, and no history of mistreatment, C-statistic: 0.73 (Table 2). In contrast, inclusion in profile D was predicted by self-identified female sex, Black race, early or mid-career burnout, and a history of mistreatment, C-statistic: 0.80.

**Table 2:** Demographic and Personal Characteristics Independently Predicting Membership in Profile A and Profile D Using Multivariable Analysis

(Second Term Is the Reference)	Predictors	
	Profile A	Profile D
No mistreatment vs mistreatment	**1.75 (1.32-2.31)**	-
Mistreatment vs no mistreatment	-	**3.95 (2.24-6.97)**
Race ethnicity (White is the reference)		
Other vs White	-	3.21 (1.37-7.51)
Black vs White	**0.107 (0.04-0.30)**	**11.92 (6.91-20.55)**
Asian vs White	0.61 (0.42-0.88)	1.59 (1.07-2.38)
Hispanic vs White	-	1.95 (1.11-3.41)
Sex		
Men vs women	**5.19 (3.44-7.84)**	-
Women vs men	--	**2.70 (1.97-3.69)**
Tenure (late career is the reference)		
Fellow in training vs late career (22+ y)	0.54 (0.34-0.86)	
Early career (1-7 y) vs late career (22+ y)	0.65 (0.43-0.98)	1.94 (1.23-3.04)
Mid (8-14 y) vs late career (22+ y)	--	2.08 (1.30-3.35)
No training information vs late career (22+ y)	0.55 (0.34-0.92)	--
Other		
No burnout vs burnout	--	--
Burnout vs no burnout	--	1.66 (1.18-2.33)
Model statistics		
Sensitivity	92.4%	96.4%
Specificity	19.0%	23.7%
Accuracy	71.8%	82.9%
C-statistic/Receiver operator curve (CI)	0.73 (0.70-0.75)	0.80 (0.77-0.83)

*P* ≤ 0.01 was threshold for model inclusion; *P* < 0.001 ORs are **bold**.

Predictors are shown as OR (95% CI).

### Perspectives on diversity and need for change within cardiology

**Figure 2:**
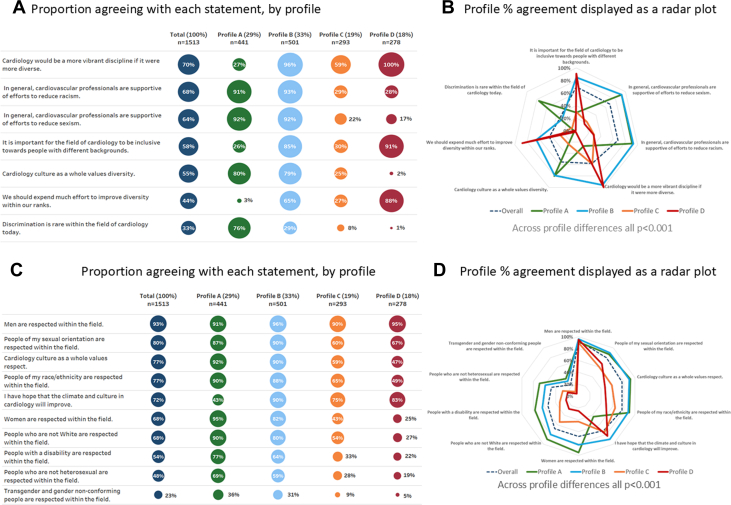

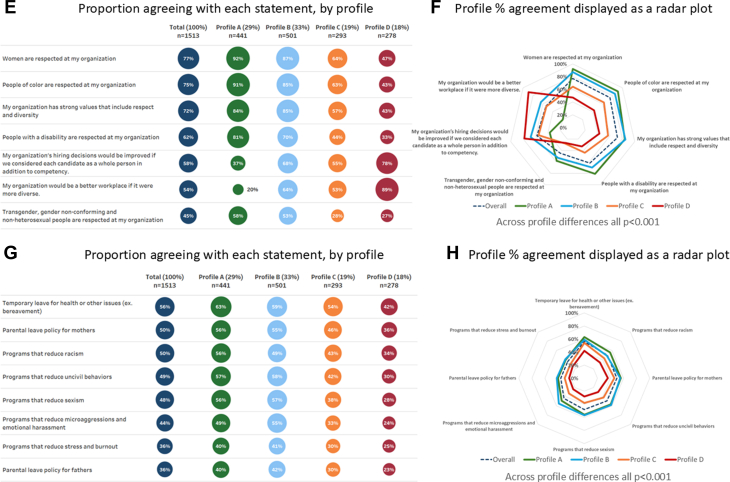
**Perceptions of Professional Climate by Profile** These paired figures show agreement with perceptions by profile for Workforce (A and B), Respect (C and D), Primary workplace climate (E and F), and Effectiveness of efforts to improve climate (G and H).

Most cardiologists (70%) felt that cardiology would be more vibrant if it were more diverse and were supportive of efforts to reduce racism (68%) and sexism (64%). There was weaker agreement that cardiology’s climate valued diversity (55%) or that inclusivity was important to the field (58%) and 33% felt that discrimination was rare (Supplemental Table 1). Less than half (44%) felt that cardiology should expend “much effort” to improve diversity. There were significant and sometimes dramatic variations across profiles in virtually all perceptions of statements related to workforce diversity and the need for an inclusive environment (Table 3, Figures 2A to 2H). Similarly, impressions of the prevalence of discrimination and support for efforts to reduce racism and sexism varied widely among profiles. While 80% of profile A and 79% of B respondents agreed that “*Cardiology culture as a whole values diversity*,” only 25% and 2% of profiles C and D, respectively, agreed (Supplemental Table 1). Inclusivity was felt to be important in cardiology by nearly all B (85%) and D (91%) respondents, but not among A and C (26% and 30%). In parallel, discrimination was felt to be rare among 76% of A, but much less so among B, C, and D respondents (29%, 8% and 1%, respectively). Profiles A and B felt that cardiology supported efforts to reduce racism: 91% and 93%; sexism 92% and 92%, in contrast to profiles C and D (racism: 29%, 28%; sexism 22%, 17%). Substantial efforts to improve diversity were not endorsed by 97% of A and 73% of C, while B and D favored such efforts (65%, 88%).

**Table 3:** Difference in Perceptions of Professional Climate in Cardiology: Diversity, Respect, Workplace Culture, and Effectiveness

	Profile A	Profile B	Profile C	Profile D
	Empowering, Inclusive Culture/No Change Needed	Empowering, Inclusive Culture/Change Needed	Stifling, Exclusionary Culture/Change Uncertain	Stifling, Exclusionary Culture/Change Needed
	(n = 441, 29%)	(n = 501, 33%)	(n = 293, 19%)	(n = 278, 18%)
Agreement with overall cardiology workforce values statements				
Cardiology would be a more vibrant discipline if it were more diverse.	Ref	**16.55 (11.95-22.93 CI)**	1.23 (0.89-1.71 CI)	**30.36 (18.98-48.56 CI)**
In general, cardiovascular professionals are supportive of efforts to reduce racism.	Ref	1.30 (0.80-2.10 CI)	**0.04 (0.03-0.06 CI)**	**0.04 (0.02-0.06 CI)**
In general, cardiovascular professionals are supportive of efforts to reduce sexism.	Ref	0.94 (0.59-1.50 CI)	**0.02 (0.02-0.04 CI)**	**0.02 (0.01-0.03 CI)**
It is important for the field of cardiology to be inclusive toward people with different backgrounds.	Ref	**67.09 (40.53-111.04 CI)**	**3.87 (2.83-5.29 CI)**	**732.56 (101.69-5277.23 CI)**
Cardiology culture as a whole values diversity.	Ref	0.95 (0.69-1.31 CI)	**0.08 (0.06-0.12 CI)**	**0.01 (0.00-0.01 CI)**
We should expend much effort to improve diversity within our ranks.	Ref	**55.35 (31.52-97.17 CI)**	**11.26 (6.23-20.34 CI)**	**226.44 (118.86-431.41 CI)**
Discrimination is rare within the field of cardiology today.	Ref	**0.13 (0.10-0.18 CI)**	**0.03 (0.02-0.05 CI)**	**0.01 (0.00-0.01 CI)**
Agreement with overall cardiology respect statements				
Men are respected within the field.	Ref	**2.17 (1.27-3.72 CI)**	0.87 (0.53-1.43 CI)	**2.03 (1.07-3.87 CI)**
People of my sexual orientation are respected within the field.	Ref	1.39 (0.93-2.08 CI)	**0.30 (0.21-0.43 CI)**	**0.32 (0.22-0.46 CI)**
Cardiology culture as a whole values respect.	Ref	0.73 (0.46-1.16 CI)	**0.12 (0.08-0.18 CI)**	**0.07 (0.05-0.11 CI)**
People of my race/ethnicity are respected within the field.	Ref	0.76 (0.50-1.15 CI)	**0.19 (0.13-0.29 CI)**	**0.10 (0.07-0.15 CI)**
I have hope that the climate and culture in cardiology will improve.	Ref	**12.41 (8.75-17.62 CI)**	**3.98 (2.88-5.51 CI)**	**6.45 (4.48-9.28 CI)**
People who are not White are respected within the field.	Ref	**0.46 (0.31-0.66 CI)**	**0.14 (0.09-0.20 CI)**	**0.04 (0.03-0.06 CI)**
Women are respected within the field.	Ref	**0.26 (0.16-0.42 CI)**	**0.04 (0.03-0.07 CI)**	**0.02 (0.01-0.03 CI)**
People with a disability are respected within the field.	Ref	**0.52 (0.39-0.69 CI)**	**0.15 (0.10-0.20 CI)**	**0.08 (0.06-0.12 CI)**
People who are not heterosexual are respected within the field.	Ref	**0.65 (0.50-0.85 CI)**	**0.18 (0.13-0.25 CI)**	**0.11 (0.07-0.15 CI)**
Transgender and sex nonconforming people are respected within the field.	Ref	0.79 (0.60-1.03 CI)	**0.18 (0.12-0.28 CI)**	**0.09 (0.05-0.16 CI)**
Agreement with local workplace climate statements				
Women are respected at my organization	Ref	**0.56 (0.36-0.88 CI)**	**0.15 (0.09-0.22 CI)**	**0.07 (0.05-0.11 CI)**
People of color are respected at my organization	Ref	**0.57 (0.38-0.86 CI)**	**0.17 (0.11-0.25 CI)**	**0.08 (0.05-0.11 CI)**
My organization has strong values that include respect and diversity	Ref	1.06 (0.74-1.50 CI)	**0.25 (0.18-0.35 CI)**	**0.15 (0.10-0.21 CI)**
People with a disability are respected at my organization	Ref	**0.55 (0.40-0.74 CI)**	**0.19 (0.13-0.26 CI)**	**0.12 (0.08-0.16 CI)**
My organization’s hiring decisions would be improved if we considered each candidate as a whole person in addition to competency.	Ref	**3.70 (2.83-4.85 CI)**	**2.07 (1.53-2.80 CI)**	**6.13 (4.34-8.64 CI)**
My organization would be a better workplace if it were more diverse.	Ref	**7.28 (5.41-9.79 CI)**	**4.51 (3.25-6.25 CI)**	**30.84 (19.94-47.70 CI)**
Transgender, sex nonconforming and nonheterosexual people are respected at my organization	Ref	0.83 (0.64-1.07 CI)	**0.28 (0.21-0.39 CI)**	**0.27 (0.20-0.38 CI)**
Perceptions of local workplace effectiveness				
Temporary leave for health or other issues (ex. bereavement)	Ref	0.82 (0.62-1.06 CI)	**0.68 (0.50-0.91 CI)**	**0.42 (0.31-0.57 CI)**
Programs that reduce racism	Ref	1.14 (0.88-1.47 CI)	**0.59 (0.44-0.80 CI)**	**0.41 (0.30-0.56 CI)**
Parental leave policy for mothers	Ref	0.96 (0.74-1.24 CI)	**0.69 (0.51-0.92 CI)**	**0.44 (0.32-0.60 CI)**
Programs that reduce uncivil behaviors	Ref	1.02 (0.79-1.32 CI)	**0.55 (0.41-0.74 CI)**	**0.32 (0.23-0.44 CI)**
Programs that reduce sexism	Ref	1.04 (0.81-1.35 CI)	**0.48 (0.36-0.65 CI)**	**0.30 (0.22-0.41 CI)**
Programs that reduce microaggressions and emotional harassment	Ref	**1.30 (1.01-1.68 CI)**	**0.52 (0.38-0.70 CI)**	**0.34 (0.24-0.47 CI)**
Parental leave policy for fathers	Ref	1.12 (0.87-1.46 CI)	**0.65 (0.48-0.89 CI)**	**0.46 (0.33-0.65 CI)**
Programs that reduce stress and burnout	Ref	1.03 (0.80-1.34 CI)	**0.64 (0.46-0.87 CI)**	**0.52 (0.37-0.72 CI)**

OR and 95% CIs by univariate logistic regression analysis using profile A’s percent agreement as the reference for comparison to other profiles. ORs below 1 mean that respondents in that profile are less likely to agree with the statement than those in profile A, while numbers above 1 are more likely to agree. **Bold** text represents significance at the *P* < 0.01 level, to correct for multiplicity.

### Perceptions of respect in the profession of cardiology

Profiles varied in agreement with statements related to respect in cardiology (Figures 2C and 2D, Supplemental Table 1, Supplemental Figure 2A), with significant differences between A and all other profiles (Table 2). Nearly all respondents (93%) in all groups agreed men were respected in cardiology (range across profiles 90%-96%). There was less overall agreement and greater variation across profiles about respect for people who identify as non-White (68%; 27%-90%), women (68%; 25%-95%), people with a disability (54%; 22%-77%), and nonheterosexual (48%; 19%-69%) or transgender/sex nonconforming people (23%; 5%-36%). In general, as with workforce diversity statements, profiles A and B strongly felt that most groups were respected in cardiology, while profile D perceived that only men and people of similar sexual orientation (the cohort was 93% heterosexual) were respected. Profile C held intermediate views.

### Perceptions of primary workplace climate

Survey participants were queried on their perceptions of professional climate at their primary workplace including aspects related to workforce diversity, inclusion, and respect and effectiveness of efforts to improve institutional climate. Agreement that diversity was valued was higher in the local workplace (72%; range 43%-84%) as compared with perceptions of the overall cardiology climate, 55%; range 2% to 80% (Figures 2E and 2F, Supplemental Table 1, Supplemental Figure 2B). Moreover, overall agreement with workplace perceptions of respect was slightly higher for most groups and profile related variation was less marked than in perceptions of the overall cardiology climate. This included respect for people who identify as Black, Asian, or Hispanic (75%; range across profiles 43%-91% vs 68% for cardiology), women (77%; 47%-92% vs 68%), people with a disability (62%; 33%-81% vs 54%), and transgender/sex nonconforming people (45%; 27%-58% vs 23%).

As with the overall cardiology climate, cardiologists aligned with profiles A and B held more positive views about their organizations’ workforce diversity values and respect for specific groups, while profiles C and especially D indicated more negative views. About half (54%; 20%-89%) felt that their workplace would be improved by more diversity, with similar wide variation in agreement that hiring decisions would be improved by a holistic approach (58%; 37%-78%).

### Perceptions of primary workplace effectiveness at improving climate

Across all respondents, half or less felt that efforts to improve climate at their primary workplace were effective, including those that reduced racism (50%; range 34%-50%), sexism (48%; range 28%-57%), uncivil behaviors (49%; 30%-58%), microaggressions and harassment (44%; 24%-55%) or reduced stress or burnout (36%; 25%-41%) (Table 3, Figures 2G and 2H, Supplemental Table 1). Leave policies were rated as effective by 50% (maternity leave) and 36% (paternity leave) of respondents. Disagreements across profiles were more muted than with other questions, with profile A and B respondents having nearly identical perspectives, with C and D progressively less likely to have positive perceptions of effectiveness. However, differences between A and D were only in the range of 10% to 20%.

### Free-text comments

Three hundred fifty-one classifiable comments were received from 284 respondents (Supplemental Table 4). The most common themes were agreement that the professional or workplace climate needed to improve (29% of comments), and reports of personal experiences of mistreatment (29%). Other commenters felt that there was too much emphasis being placed on diversity, equity, and inclusion (10%), that merit was more important than diversity (10%), that progress is being made on diversity (9%) and gratitude for ACC’s DEI efforts (7%). Fifteen (4%) comments stated that they had never witnessed or experienced discrimination. Examples of comments from respondents in each profile are shown in Supplemental Table 2.

## Discussion

The culture of medicine and its translation into the perceptions and lived experiences, or climate, are closely related to individual and workforce well-being and patient outcomes. In this national survey of professional climate among U.S. cardiologists and trainees, we found that 37% felt that cardiology has a stifling and exclusionary climate and over half supported substantial efforts to increase inclusion. Only 29% thought no change was necessary. At its extremes, this heterogeneity could be predicted with good accuracy by a combination of respondents’ demographics and other personal characteristics. Although dissatisfaction was more muted at the local institutional level, most respondents felt that efforts to improve the climate at their primary workplace were ineffective. Understanding cardiologists' work environment is vital for designing strategies that enhance their well-being and positively influence patient care.

We report a comprehensive assessment of perceptions of professional climate through the lens of U.S. cardiologists and trainees. It differs substantially from prior surveys in several important ways. First, some prior surveys in cardiology and beyond, have highlighted negative personal experiences, or mistreatment, and have not always captured positive impressions of the lived experience in cardiology.^4^ Second, prior surveys have not fully captured the unique insights of those groups historically underrepresented in cardiology and cardiology leadership, or the impact of identity intersectionality. Third, rather than analyzing across all respondents to present an “average” view which would minimize and obscure interpersonal and intergroup differences, our analysis created profiles of respondents with similar views which captures the range of perceptions and gives a voice to those with positive as well as negative views. Fourth, this person-centered approach differs substantially from the usual variable-centered one, by focusing on the perceptions themselves and not the identity groups which may hold them (eg, men vs women). It allows a multifaceted picture of climate, including possibly unrelated perceptions. This approach, sometimes termed profile creation, has proven valuable in the psychological literature in understanding burnout in medical professionals and work fatigue.^20^, ^21^, ^22^ In the biological sciences, it is somewhat analogous to Phenome Wide Association Studies (as distinct from Genome Wide Association Studies) which determine the genetic underpinnings of a constellation of multiple traits rather presence or absence of a single disease.^23^ Finally, and perhaps most importantly, identification of groups of individuals with similar perceptions can help guide the design of interventions to improve climate and ensure that they are tailored to individual needs.

The importance of this novel approach is reflected in our data, which shows that while perceptions can be averaged across all profiles to indicate the state of cardiology as a whole, these numbers are not informative or representative, as shown graphically in the radar plots (Figures 2B, 2D, 2F, and 2H). This is critically important as profile A respondents differed significantly from the other 3 profiles in their perceptions of virtually all climate statements (Table 3), expressing positive views that may be overlooked in reports focused on more memorable negative views or experiences. Similarly, strong negative feelings about diversity might otherwise go unheard among the larger number of supportive responses. This is also true for ensuring a voice for those aligned with profile D: their highly negative views of climate and positive feelings about diversity would be lost in an overall analysis. This group is of particular importance as they may be at highest risk for disengagement and even leaving the profession, and younger individuals are over represented. Between these 2 extremes, profile B represents those who are satisfied with the current climate but still feel that it can be improved further, while profile C is dissatisfied with the climate yet appears uncertain about the need for change.

### Identities and profile alignment

While all demographic groups are represented to some extent in all profiles, there were clear tendencies in profile membership across multiple demographic and personal characteristics. Furthermore, characteristics of race and ethnicity, sex, experience of mistreatment, and career stage independently predicted membership in both profiles A and D with good to excellent accuracy. However, exploration of intersectionality of 2 of these: sex and race and ethnicity, reveals that analysis by either one alone would conceal important differences, including the “double jeopardy” experienced by underrepresented women amplified in Black women.^24^ White men were most likely to be in A, while Asian men were more likely to be in B. Most Hispanic men were either in A or B while Black men were almost exclusively in D. Alignment with profile A was rare for women of any race or ethnicity. Hispanic women straddled B and C, with White and Asian women equally split between B, C, and D. Black women were most likely to be in D. Higher proportions of younger individuals were in profiles C and D, especially compared to the most senior, who were more likely to align with profile A views. Although examination by sex is common, most prior studies of culture and climate do not report results with this level of attention to multiple specific groups, and the substantial impact of intersectionality is largely unexamined.^10^^,^^25^

### Mistreatment and burnout and climate perceptions

Respondents reporting burnout make up higher proportions of profiles C and D than A or B (see column % data in text and Supplemental Tables 2 and 3). This is aligned with prior reports in cardiology showing significant prevalence of burnout, and with primary care physicians which show strong associations between burnout and workplace climate, especially workload, organization satisfaction and support, and connection to mission.^2^^,^^3^^,^^14^^,^^26^ In our study, the majority of those reporting mistreatment and half of those with burnout are aligned with profiles A and B (row % data as shown in Table 1). This suggests that alignment with profiles C and D is not solely due to negative personal experience despite the higher prevalence of mistreatment reported by these groups. Nevertheless, there is likely some interplay, although it would be tremendously difficult to parse causation.

### Respect and cultural change

Across profiles, there is strong consensus that men are respected. This is not true for any other demographic or personal identity group. Profile A felt that most groups are respected (except those with underrepresented sexual and sex identities) and profile D felt that no other groups are respected. Although half the respondents favored change, including those satisfied with the current climate (the plurality aligned with profile B), the significant challenges of cultural transformation within cardiology are amplified by these divergent attitudinal perceptions.

### Comparison to other surveys

To our knowledge, this is the first detailed survey on broader aspects of climate and culture within cardiology and the first to use a person-centered, profile analysis approach. The scarcity of comparative national data is exacerbated by the frequent performance of organizational climate surveys as internal quality improvement initiatives, not intended for publication as research. Furthermore, survey instruments are not uniform and may lack the granularity of our data.

A commonly used culture survey instrument in academic medicine is the C-Change survey, which has been validated for use among faculty, residents, and students.^27^ The European Society of Cardiology utilized the C-Change survey to query members in 17 European countries, uncovering sex differences in perceptions of culture and sex equity, which were overshadowed by regional differences. These results are unpublished.^28^ Faculty survey data at UC San Diego Health Sciences included questions on climate and faculty behavior, however publicly available data do not include demographics beyond sex and rank, or exact questions.^29^ Several recent physician surveys reported lower intent to leave one’s current organization associated with feelings of belonging, or feeling a valued member of a group or organization.^30^, ^31^, ^32^ Since this is more related to institutional culture and leadership than interpersonal relationships, the low perceived organizational effectiveness among our respondents is an especial concern. This may be reflected in profile C, who had high climate concerns but were uncertain about expending much effort to enhance inclusion. Their negative views may be related to limited support, poor leadership, and not feeling valued rather than diversity. Other professions have performed similar surveys, included the American Economic Association whose 2019 survey noted that 34% of respondents were “*satisfied with the overall climate within the field of economics”* while 56% were “*satisfied with the overall climate at my institution/place of employment*.” Responses varied by age, sex, disability, religion, and sexual orientation. These data, while from a field remote to cardiology, underscore the importance of directly evaluating lived experience within a profession, including comparison of multiple demographic and other groups and overall and local climate.^12^

### Using climate data to improve cardiologists’ professional experience

While our descriptions of the climate perceptions of cardiology are novel and of interest, their greatest value lies in guiding improvements in the lived experiences of cardiologists and trainees. Our data quantify the degree of resistance (or acceptance) of change among cardiologists and indicates their level of engagement (or opposition) to culture and climate initiatives which are essential to guiding strategic efforts to battle 2 major crises in cardiology: workforce and clinician well-being, both with significant business and health care implications. Along these lines, Wingard et al detail a road map for using serial climate surveys to successfully drive workforce program and policy changes at an academic medical center, which resulted in improvement in culture and climate.^33^ Following this approach, faculty felt better informed and that they had more adequate and equitable resources including research space and access to mentoring. Furthermore, negative faculty behaviors were reduced, including derogatory comments, diminished work productivity, anger outburst, and hostile communication.^33^ Other authors have noted that understanding climate is essential to realizing the numerous benefits brought by a diverse, inclusive culture, including better health outcomes, reduced health disparities, and higher impact science.^34^, ^35^, ^36^, ^37^ Based on their review of 17 studies of success factors in academic medicine, Vassie et al emphasized the importance of considering “*structural and cultural factors as well as individual needs”* when addressing the clinical workplace, along with other “*more standard good practices”* to enhance diversity and equity such as family friendly policies, transparent career pathways, and mitigating interpersonal conflict.^38^

Our findings provide granular support for continued targeted interventions and are essential to enhancing the effectiveness of efforts to improve cardiovascular educational, research, and practice environments. The inadequacy of current efforts to “listen” to climate is suggested by the consensus of limited institutional effectiveness, including programs that reduce racism, sexism, uncivil behaviors, microaggressions and emotional harassment, or stress and burnout, as well as maternity and paternity leave policies. Clearly, these well-meaning workplace efforts, stimulated by a desire for a positive culture, were not widely appreciated as contributing to a positive lived experience. This is an important lesson for leadership about the need to be attentive to climate as a personal commitment may not translate into cardiologists’ positive lived experience.^39^ Additional strategies to improve climate include focused training/education of those with the greatest responsibility for setting and guiding the culture in individual institutions/health systems and access to specific tools to measure well-being, burnout, and overall perception of culture. Finally, survey data should be included in devising and implementing organizational programs.

### Study Limitations

While our study takes a unique person-centered approach and the results are highly novel, culture and climate are profoundly complex concepts and there are no best practices or accepted measures for evaluating them.^40^ The subjective nature of perceptions of climate and their variation depending on many factors are fundamental principles underpinning our study. Similarly, it is challenging to offer a single or central view of the climate of cardiology as it is situational and often includes complex dimensions such as autonomy, support, and leadership, along with more objective items such as workload and compensation, some of which were beyond the scope of our survey. Analysis of all potential dimensions is not possible in one study. Although SPSS Two-Step Cluster Analysis is useful for exploring natural groupings in large data sets, it has some limitations. The method offers limited control over clustering parameters and lacks transparency in the preclustering phase, reducing reproducibility. The initial preclustering step is sensitive to the order in which the data are presented and analysis on a reordered data set can produce a different cluster model. Additionally, diagnostic and validation tools are limited to silhouette coefficients to assess cluster quality.

As with any modeling, cluster creation or the related profile analysis may be imperfect.^13^ However, the goal was to generate profiles with clear separations of responses to give voice to and recognize different perceptions of climate in cardiology. This was accomplished. In fact, the striking differentiation between profiles in their perceptions and the ability to predict alignment of views strongly supports the validity and value of our profile creation approach and methodology.

Our response rate was similar to other ACC surveys focusing on nonclinical issues, which are also ∼10%. The concern that the resulting sample may not be representative is mitigated by our sending the survey to a wide range of U.S. cardiologists, and by the similarity of respondent demographics to those surveyed and to demographic data across all of cardiology. However, we cannot eliminate the potential for response bias. Unfortunately, the analytic approach of cluster creation based on a matrix of responses is not amenable to results weighting. Even among respondents, some cardiologists may be reluctant (or over eager) to disclose their negative views, or their demographic information. Finally, our findings represent a snapshot of cardiologists’ perceptions about climate and culture in the United States at the time of our survey and may not reflect professional climates and cardiologists’ perceptions outside the United States or changes over time. Larger studies using a similar person-centered approach, and ideally repeated over time, would help to more fully understand the cardiology climate, factors are associated with positive vs negative views and how best to use these data to improve cardiologists’ professional lives.

## Conclusions

In this national survey of cardiologists’ perceptions, cardiologists and trainees hold divergent views of their professional climate which are differentiated by far more than demographic variables. Nevertheless, a significant proportion (37%) of our mostly White, mostly male respondents perceived the culture and climate in cardiology as stifling/exclusionary and half desired change (51%). It is important to recognize that, in contrast, 29% did not favor expending effort on increasing inclusion. These findings, plus perceptions of organizational ineffectiveness, support a call to action to improve the cardiology climate and identify a need to incorporate climate assessments in national and local efforts to address critical challenges. Fostering an inclusive and civil climate in cardiology requires systematic action to ensure that positive values are more closely translated into a positive lived experience among the workforce, improved well-being, and optimal patient care.

## Funding support and author disclosures

The authors have reported that they have no relationships relevant to the contents of this paper to disclose.

## Supplementary Materials

Supplemental Table s1 to s4 and Supplemental Figures s1 to s3
