# Supplementary material for: Spectrum of Perceptions Regarding Professional Climate: A National Survey of U.S. Cardiologists and Trainees
Source: JACC Adv. 2025 Nov 15;4(12):102340. doi: 10.1016/j.jacadv.2025.102340 (PMC12664046; doi:10.1016/j.jacadv.2025.102340)

Supplementary files

Spectrum of Perceptions Regarding Professional Climate: A National Survey of US Cardiologists and Trainees

Supplementary Tables:

Supplemental Table s1. Perceptions of professional climate in cardiology related to diversity, respect, overall and local workplace culture and effectiveness of local efforts to improve climate

Supplemental Table s2: Experiences of mistreatment in clinical and academic settings by profile

Supplemental Table s3: Career impact of mistreatment by profile

Supplemental Table s4: Responses to open ended question

Supplementary Figures:

Supplemental Figure s1: Cluster Creation Inputs

Supplemental Figure s2: Results of the Profile Creation Model

Supplemental Figure s3A: Perceptions of Professional Climate in Cardiology: Respect for Specific Groups

Supplemental Figure s3B: Perceptions of Climate at Primary Workplace: Respect for Specific Groups

Supplemental Table s1. Perceptions of professional climate in cardiology related to diversity, respect, overall and local workplace culture and effectiveness of local efforts to improve climate

	All respondents	Profile A	Profile B	Profile C	Profile D	
**Profile description**		Empowering , Inclusive Culture/ No change needed	Empowering , Inclusive Culture/ Change needed	Stifling, Exclusionary Culture/ Change uncertain	Stifling, Exclusionary Culture/Cha nge needed	P value for intergroup differences
**N, %**	N=1513 100%	N=441 29%	N=501 33%	N=293 19%	N=278 18%	
**Perceptions of Workforce (Strongly agree/agree)**						
Cardiology would be a more vibrant discipline if it were more diverse.	1054, 70%	27%	96%	59%	100%	<.0.001
In general, cardiovascular professionals are supportive of efforts to reduce racism.	1032, 68%	91%	93%	29%	28%	<.0.001
In general, cardiovascular professionals are supportive of efforts to reduce sexism.	975, 64%	92%	92%	22%	17%	<.0.001
It is important for the field of cardiology to be inclusive towards people with different backgrounds.*	883, 58%	26%	85%	30%	91%	<.0.001
Cardiology culture as a whole values diversity.	833, 55%	80%	79%	25%	2%	<.0.001
We should expend much effort to improve diversity within our ranks.*	661, 44%	3%	65%	27%	88%	<.0.001
Discrimination is rare within the field of cardiology today.	506, 33%	76%	29%	8%	1%	<.0.001
**Perceptions of Respect (Strongly agree/agree)**						
Men are respected within the field.	1408, 93%	91%	96%	90%	95%	<.0.001
People of my sexual orientation are respected within the field.	1213, 80%	87%	90%	60%	67%	<.0.001
Cardiology culture as a whole values respect.	1165, 77%	92%	90%	59%	47%	<.0.001
People of my race/ethnicity are respected within the field.	1164, 77%	90%	88%	65%	49%	<.0.001
I have hope that the climate and culture in cardiology will improve.	1089, 72%	43%	90%	75%	83%	<.0.001
People who are not White are respected within the field.	1026, 68%	90%	80%	54%	27%	<.0.001
Women are respected within the field.	1026, 68%	95%	82%	43%	25%	<.0.001
People with a disability are respected within the field.	815, 54%	77%	64%	33%	22%	<.0.001
People who are not heterosexual are respected within the field.	732, 48%	69%	59%	28%	19%	<.0.001
Transgender and gender non-conforming people are respected within the field.	353, 23%	36%	31%	9%	5%	<.0.001
**Perceptions of Workplace Climate (Strongly agree/agree)**						
Women are respected at my organization	1164, 77%	92%	87%	64%	47%	<.0.001
People of color are respected at my organization	1127, 75%	91%	85%	63%	43%	<.0.001
My organization has strong values that include respect and diversity	1083, 72%	84%	85%	57%	43%	<.0.001

People with a disability are respected at my organization	932, 62%	81%	70%	44%	33%	<.0.001
My organization’s hiring decisions would be improved if we considered each candidate as a whole person in addition to competency.	881, 58%	37%	68%	55%	78%	<.0.001
My organization would be a better workplace if it were more diverse.	812, 54%	20%	64%	53%	89%	<.0.001
Transgender, gender non-conforming and non-heterosexual people are respected at my organization	679, 45%	58%	53%	28%	27%	<.0.001
**Perceptions of Workplace Effectiveness (Very effective/effective)**						
Temporary leave for health or other issues (ex. bereavement)	849, 56%	63%	59%	54%	42%	<.0.001
Programs that reduce racism	761, 50%	56%	49%	43%	34%	<.0.001
Parental leave policy for mothers	755, 50%	56%	55%	46%	36%	<.0.001
Programs that reduce uncivil behaviors	748, 49%	57%	58%	42%	30%	<.0.001
Programs that reduce sexism	724, 48%	56%	57%	38%	28%	<.0.001
Programs that reduce microaggressions and emotional harassment	659, 44%	49%	55%	33%	24%	<.0.001
Parental leave policy for fathers	541, 36%	40%	42%	30%	23%	<.0.001
Programs that reduce stress and burnout	538, 36%	40%	41%	30%	25%	<.0.001

*These statements were originally phrased as negative but inverted to positive to provide directional consistency across statements. To be conservative, the numbers shown reflect the proportion of the original ‘strongly disagree’ perceptions, now ‘strongly agree’ after the inversion.

Supplemental Table s2: Experiences of mistreatment in clinical and academic settings by profile

	All respondents	Profile A	Profile B	Profile C	Profile D	
**Profile description**		Empowering, Inclusive Culture/ No change needed	Empowering, Inclusive Culture/ Change needed	Stifling, Exclusionary Culture/ Change uncertain	Stifling, Exclusionary Culture/Change needed	P value for intergroup differences
**N, %**	N=1513 100%	N=441 29%	N=501 33%	N=293 19%	N=278 18%	
**Clinical Type of Work**						
N size	1436, 100%	424, 100%	486, 100%	262, 100%	264, 100%	
No discrimination or unfair treatment	637, 44%	59%	51%	34%	19%	<.0.001
**Total: Professional Advancement**	442, 31%	18%	25%	38%	55%	<.0.001
Promotion or advancement of self or peers	277, 19%	10%	15%	24%	38%	<.0.001
Clinical leadership roles (lab director, etc)	262, 18%	10%	15%	27%	29%	<.0.001
Invitations to participate in professional development activities	161, 11%	5%	9%	15%	22%	<.0.001
Invitations to participate in formal or informal networks	164, 11%	5%	6%	18%	26%	<.0.001
**Total: Clinical Work Expectations**	368, 26%	16%	20%	34%	43%	<.0.001
Clinical obligations	197, 14%	8%	10%	19%	26%	<.0.001
Clinical outreach or offsite activities or on call schedules	131, 9%	5%	6%	13%	18%	<.0.001
Access to clinical resources (staff procedure lab time, clinical space, etc)	148, 10%	5%	9%	12%	20%	<.0.001
Access to high RVU activities	162, 11%	7%	8%	15%	21%	<.0.001
Access to leave time	62, 4%	2%	3%	6%	9%	<.0.001
**Total: Hiring**	291, 20%	11%	16%	27%	36%	<.0.001
Fellow or trainee selection decisions	167, 12%	6%	10%	13%	22%	<.0.001
Hiring of self, colleagues or peers	185, 13%	7%	9%	20%	24%	<.0.001
**Compensation for Clinical Work**	319, 22%	12%	20%	25%	40%	<.0.001
**Academic Type of Work**						
N size	844, 100%	208, 100%	317, 100%	144, 100%	175, 100%	
No discrimination or unfair treatment	361, 43%	54%	51%	29%	26%	<.0.001
**Total: Professional Advancement**	186, 22%	14%	18%	29%	34%	<.0.001
Access to leadership roles	157, 19%	11%	13%	26%	31%	<.0.001
Invitations for speaking roles and/or writing an editorial	97, 12%	8%	8%	14%	21%	<.0.001
**Total: Compensation**	186, 22%	20%	17%	23%	32%	.0.002
Compensation for academic work	164, 19%	17%	16%	22%	26%	0.038
Funding decisions	58, 7%	7%	5%	5%	12%	0.020
**Total: Access To Research Opportunities**	184, 22%	11%	18%	29%	35%	<.0.001
Access to potential coauthors or potential collaborators	97, 12%	6%	9%	15%	21%	<.0.001
Access to research-related resources (support and research personnel, space, costs)	120, 14%	6%	12%	19%	25%	<.0.001
Access to student or trainee researchers	61, 7%	4%	6%	7%	13%	0.004
Access to data	65, 8%	4%	6%	10%	13%	<.0.001
**Total: Publishing Role**	152, 18%	12%	13%	25%	29%	<.0.001
Inclusion as a coinvestigator or coauthor	105, 13%	8%	10%	19%	19%	<.0.001
Author position on papers	86, 10%	5%	7%	13%	21%	<.0.001
Publishing decisions	40, 5%	4%	4%	4%	9%	0.023

Supplemental Table s3: Career impact of mistreatment by profile

	All respondents	Profile A	Profile B	Profile C	Profile D	
**Profile description**		Empowering, Inclusive Culture/ No change needed	Empowering, Inclusive Culture/ Change needed	Stifling, Exclusionary Culture/ Change uncertain	Stifling, Exclusionary Culture/Change needed	P value for intergroup differences
**N, %**	N=1513 100%	N=441 29%	N=501 33%	N=293 19%	N=278 18%	
**ACTIONS TAKEN TO AVOID HARASSMENT/DISCRIMINATION**						
None	881, 58%	77%	63%	48%	32%	<.0.001
**Total: Avoided Training, Employment or Promotion**	301, 20%	11%	16%	24%	37%	<.0.001
Not applied or not accepted admission to training program or employment	91, 6%	2%	4%	6%	15%	<.0.001
Not applied or not taken a promotion or more visible role at place of employment	97, 6%	4%	5%	6%	13%	<.0.001
Left a particular employment position	186, 12%	7%	9%	18%	22%	<.0.001
**Total: Silenced**	323, 21%	10%	16%	29%	42%	<.0.001
Not presented question, idea, or view at your organization	295, 20%	8%	15%	26%	38%	<.0.001
Changed the content, method, or conclusions of a research paper	12, 1%	<1%	<1%	1%	2%	0.019
Not started or continued research in a particular area of cardiology	72, 5%	2%	3%	7%	10%	<.0.001
**Total: Social Avoidance**	247, 16%	7%	12%	25%	31%	<.0.001
Not attended in a regional or national conference	66, 4%	1%	3%	6%	9%	<.0.001
Not spoken at a regional or national conference or during a presentation	52, 3%	2%	2%	5%	7%	<.0.001
Not attended social events at work, after hours or at a conference	207, 14%	6%	9%	21%	27%	<.0.001
**OUTCOME** (N size)	743, 100%	136, 100%	226, 100%	173, 100%	208, 100%	
Nothing	275, 37%	39%	35%	40%	36%	0.699
Be less productive or effective in your work	159, 21%	21%	20%	23%	22%	0.920
**Total: File Charges or Consider Legal Action**	80, 11%	9%	11%	11%	12%	0.875
File official charge of complaint with your employer	47, 6%	4%	6%	7%	7%	0.745
Consider taking legal action	46, 6%	5%	8%	6%	5%	0.742
**Total: Consider Avoiding Work, Position, or Meetings**	303, 41%	38%	39%	42%	44%	0.684
Consider leaving a project, committee, program, or other similar work endeavor	105, 14%	13%	13%	15%	15%	0.845
Consider leaving your position	234, 32%	32%	30%	32%	32%	0.956
Take leave, sick time, miss work unexpectedly, or other similar time away from work	15, 2%	2%	1%	2%	3%	0.308
Consider not attending future cardiology meetings or conferences	66, 9%	9%	8%	8%	11%	0.604
**Total: Consider Leaving the Profession**	82, 11%	10%	8%	15%	13%	0.173
Consider leaving the field of cardiology	63, 9%	4%	7%	13%	10%	0.021
Consider leaving medicine entirely	51, 7%	8%	5%	8%	8%	0.559

Supplemental Table s4: Responses to open ended question

The survey asked: "Do you have any comments or ideas to share about the climate of the field of cardiology as it relates to diversity, inclusion, harassment, and professional conduct?

A. Overall response: 351 classifiable comments from 284 commentors

B. Types of comments (in descending order of frequency):
More work on workplace climate needed: 103 (29%)
Personal experiences of discrimination: 101 (29%)
Too much emphasis on DEI: 35 (10%)
Merit more important than DEI: 35 (10%)
Progress is being made on diversity: 31 (9%)
Appreciate ACC's efforts on DEI: 23 (7%)
Never witnessed/experienced discrimination: 15 (4%)
Other: 8

C. Examples of verbatim responses from each profile:

**Profile A**	**Profile B**	**Profile C**	**Profile D**
Empowering, Inclusive Culture / No change needed	Empowering, Inclusive Culture/ Change needed	Stifling, Exclusionary Culture/ Change indifference	Stifling, Exclusionary Culture/Change needed
113 comments 90 commentors	76 comments 66 commentors	76 comments 63 commentors	86 comments 65 commentors
Success and respect in cardiology is ability driven, and measured by quality of work, integrity, and professional behavior. I really have not seen colleagues mistreated based on gender, race or sexual orientation.	I feel there has been improvement in these areas through the years. Awareness and continued efforts necessary	Cardiology employs a wide variety of humans, and it is hard to imagine that bias, racism or sexism has anything to do specifically with Cardiology but rather the backgrounds and intrinsic weaknesses of the culture that one is raised in or adapts to- bricklayer or cardiologist.	I think that there are many cardiologists who do not think that there is a problem - ie they don't think cardiology needs to be more diverse and don't think that harassment and unprofessional conduct should be stopped.
Selection should be based on competence, not racial or sexual identity.	Cuts both ways. Discrimination exists and limits some minorities but in addition, the push to increase diversity at times discriminates against those who are not minorities.	It is all very blatant but subtle at the same time.	There is a lot of history and a lot of people in leadership or powerful positions or with powerful reputations who just don't get it and don't seem to value diversity, equity, and inclusion.
Oversensitivity leads to cancel culture. It's time to get back to science and optimal patient care.	The field of Cardiology is quite diverse and continued commitment to increase diversity and inclusion will only take this further	My personal observations and experience have been that the most overt events that I have personally witnessed were related to bias related to politics, to differences in religious beliefs, and to antisemitism.	Cardiology as a specialty is still a very unhealthy environment for women and people of color. We are far from making this specialty diverse, equitable and transparent for all.

Supplemental Figure Legends

Supplemental Figure 2: Cluster Creation Inputs

This figure shows the 7 statements which formed the inputs to the cluster creation model, with responses by cluster. The blue shading shows the input importance as a predictor of cluster membership.

Supplemental Figure 2: Results of the Profile Creation Model

This figure shows the Model summary (A), Profile quality (B), and Contribution of each workforce statement to the profile model (C). As described in the Methods, a two-step cluster analysis was used to identify optimal cluster groupings by running pre-clustering first using K means and then by running hierarchical log-likelihood methods to assign individuals to clusters. https://spssanalysis.com/two-step-cluster-analysis-in-spss/ The best fitting model with the greatest discrimination between clusters yielded 4 profiles with a silhouette coefficient of 0.4 indicating fair cohesion and separation.

Supplemental Figure 3 A and B: Perceptions of Professional Climate in Cardiology: Respect for Specific Groups

A. Perceptions of respect in cardiology overall by respondents' demographic groups
B. Perceptions of respect in primary workplace by respondents' demographic groups. Note that respect for different groups in these two settings was not queried identically.

Supplemental Figure s1: Cluster Creation Inputs

Input (Predictor) Importance: 1.0, 0.8, 0.6, 0.4, 0.2, 0.0

Cluster	3	4	2	1	
Label	Profile B	Profile A	Profile C	Profile D	
Description	Empowering, Inclusive Culture/ Change needed	Empowering, Inclusive Culture/ No change needed	Stifling, Exclusionary Culture/ Change uncertain	Stifling, Exclusionary Culture/ Change needed	
Size	33.1% (501)	29.1% (441)	19.4% (293)	18.4% (278)	
Inputs	Strongly agree (64.5%)	Agree (33.6%)	Agree (53.2%)	Strongly agree (88.1%)	We should expend much effort to improve diversity in our ranks.
	Agree (64.3%)	Agree (58.7%)	Neither agree nor disagree (38.2%)	Disagree (39.2%)	In general, cardiovascular professionals are supportive of efforts to reduce sexism.
	Agree (54.9%)	Agree (53.1%)	Neither agree nor disagree (42.0%)	Disagree (53.2%)	Cardiology culture as a whole values diversity.
	Strongly agree (58.7%)	Neither agree nor disagree (46.0%)	Agree (40.3%)	Strongly agree (86.0%)	Cardiology would be a more vibrant discipline if it were more diverse.
	Disagree (41.3%)	Agree (48.3%)	Disagree (54.9%)	Disagree (53.2%)	Discrimination is rare withing the field of cardiology today.
	Agree (57.5%)	Agree (51.7%)	Neither agree nor disagree (45.4%)	Neither agree nor disagree (35.3%)	In general, cardiovascular professionals are supportive of efforts to reduce racism.
	Strongly agree (85.2%)	Agree (45.6%)	Agree (39.2%)	Strongly agree (91.4%)	It is important for the field of cardiology to be inclusive towards people with different backgrounds.

Supplemental Figure s2: Results of the Profile Creation Model

Model Summary

Algorithm	TwoStep
Inputs	7
Profiles	4

Profile Quality

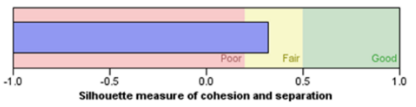

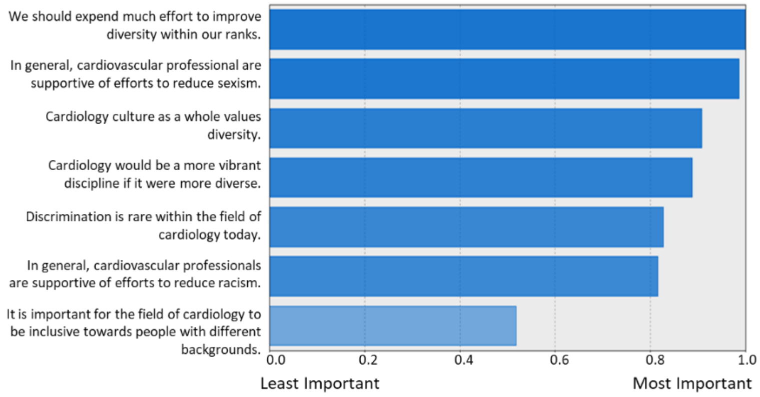

Supplemental Figure s3B: Perceptions of Climate at Primary Workplace: Respect for Specific Groups

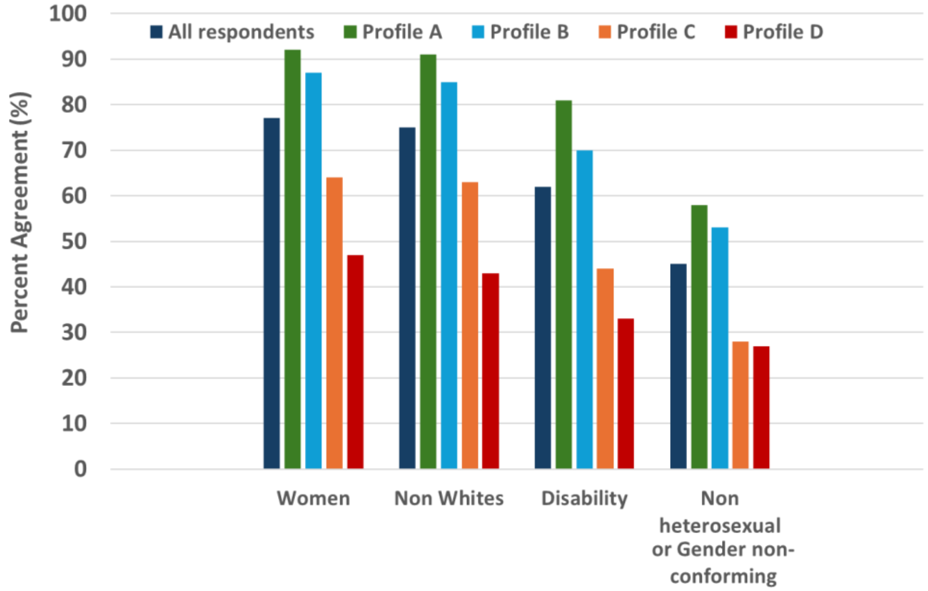

Supplemental Figure s3A: Perceptions of Professional Climate in Cardiology: Respect for Specific Groups

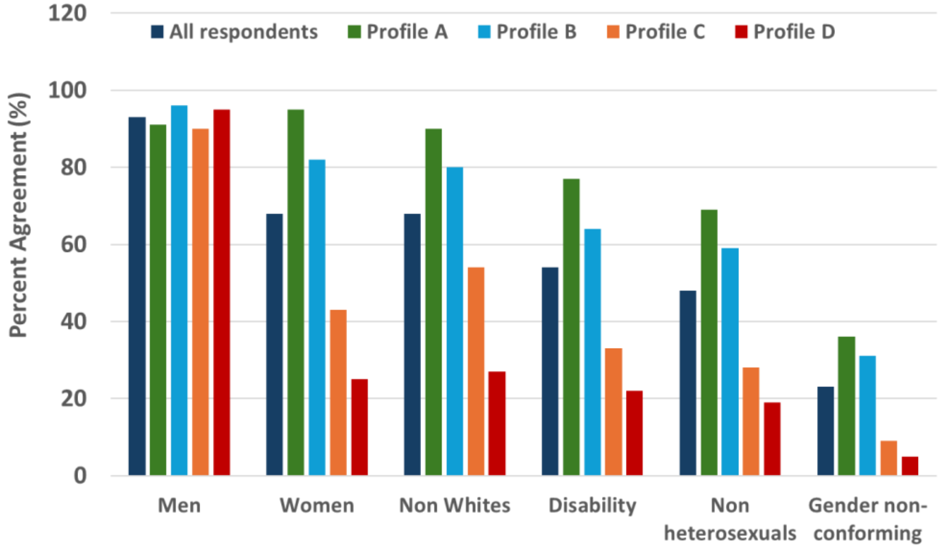

Supplement: Supplemental Table s1 to s4 and Supplemental Figures s1 to s3 [file mmc1.pdf]
